# Population-based laboratory surveillance of *Hafnia alvei *isolates in a large Canadian health region

**DOI:** 10.1186/1476-0711-5-12

**Published:** 2006-05-18

**Authors:** Kevin B Laupland, Deirdre L Church, Terry Ross, Johann DD Pitout

**Affiliations:** 1 Department of Medicine, University of Calgary and Calgary Health Region, Calgary Alberta, Canada; 2 Department of Pathology and Laboratory Medicine, University of Calgary and Calgary Health Region, Calgary Alberta, Canada; 3 Department of Critical Care Medicine, University of Calgary and Calgary Health Region, Calgary Alberta, Canada; 4 Division of Microbiology, Calgary Laboratory Services, Calgary, Alberta, Canada; 5 Centre for Anti-microbial Resistance, University of Calgary, Calgary Health Region, and Calgary Laboratory Services, Calgary, Alberta, Canada

## Abstract

**Background:**

Hospital-based series have characterized *Hafnia alvei *primarily as an infrequent agent of polymicrobial nosocomial infections in males with underlying illness.

**Methods:**

We conducted population-based laboratory surveillance in the Calgary Health Region during 2000–2005 to define the incidence, demographic risk factors for acquisition, and anti-microbial susceptibilities of *Hafnia alvei *isolates.

**Results:**

A total of 138 patients with *Hafnia alvei *isolates were identified (2.1/100,000/year) and two-thirds were of community onset. Older age and female gender were important risk factors for acquisition. The most common focus of isolation was urine in 112 (81%), followed by lower respiratory tract in 10 (7%), and soft tissue in 5 (4%), and the majority (94; 68%) were mono-microbial. Most isolates were resistant to ampicillin (111;80%), cephalothin (106; 77%), amoxicillin/clavulanate (98; 71%), and cefazolin (95; 69%) but none to imipenem or ciprofloxacin.

**Conclusion:**

*Hafnia alvei *was most commonly isolated as a mono-microbial etiology from the urinary tract in women from the community. This study highlights the importance of population-based studies in accurately defining the epidemiology of an infectious disease.

## Introduction

*Hafnia alvei *has been recognized as an infrequent cause of human infections [1]. A number of case reports and series have identified this organism as an etiologic agent of urogenital, lower respiratory tract, intra-abdominal, and skin and soft tissue infections as well as a cause of endopthalmitis and bacteremia without focus [2-6]. In the largest previously reported clinical series to date, Gunthard and Pennekamp reviewed 61 patients at two hospitals in Zurich, Switzerland [2]. They found that in three quarters of patients *Hafnia alvei *was isolated concurrently with other bacteria, most patients were male, and that the majority of isolates were associated with a respiratory tract focus. Limited data exists investigating *Hafnia alvei *as an agent of disease in the community [3-5].

Because clinical series reported from selected clinics or hospitals are potentially highly subject to selection bias, they may provide misleading information regarding the epidemiology of an infectious disease. Population-based studies, where all cases of disease occurring in a defined geographic region are studied, reduce this bias [7]. We therefore conducted a population-based laboratory surveillance study of all extra-intestinal *Hafnia alvei *isolates in a large Canadian health region to define their incidence, associated patient demographic risk factors, and microbiology.

## Methods

### Study population

The Calgary Health Region (CHR) provides all publicly funded healthcare services to the more than one million population of the cities of Calgary and Airdre and numerous adjacent surrounding communities covering an area of 37,000 km^2 ^[8]. Acute care is provided principally through one pediatric and three major adult hospitals. Nearly all (>95%) standard microbiology testing from both community and hospital sites in the CHR is performed by Calgary Laboratory Services (CLS). All patients with *Hafnia alvei *isolates identified at hospital and community based collection sites within the CHR between January 1, 2000 and December 31, 2005 were included in this study. Because detailed clinical records were not reviewed, individual written informed consent was not obtained. For purposes of this study, patients were assumed to be CHR residents and included in analysis if they were outpatients with Alberta Personal Healthcare numbers or if they were admitted to a CHR acute care facility.

### Population-based surveillance

Active, population-based surveillance for all *Hafnia alvei *isolates obtained from patients in the CHR during the study was performed by CLS. Hospitals, nursing homes, physicians' offices, and community collection sites were included in surveillance. Once *Hafnia alvei *isolates were identified, basic laboratory and demographic information was obtained from the regional laboratory information system (PathNet Classic version 306, Cerner, Kansas City, MO). Community isolates were classified as those submitted from community based collection sites or those within the first two days of admission to an acute care facility; hospital isolates were those first isolated more than two calendar days after hospital admission.

### Clinical laboratory testing

*Hafnia alvei *was isolated using standard techniques; identification and susceptibilities to anti-microbial agents were determined using Vitek (Vitek AMS; bioMérieux Vitek Systems Inc., Hazelwood, MO.). Isolates were classified as to a primary focus of infection based on the available microbiology data. Patients with cultures from either sputum, endotracheal aspirates, or bronchoalveolar lavage specimens were classified as lower respiratory foci, urine cultures greater or equal to 10^7 ^cfu/L as urinary tract, wounds or tissue specimens as soft tissue, and peritoneal fluid or abdominal operative specimens as intra-abdominal foci. Bacteremia was defined by the isolation of *Hafnia alvei *from at least one set of blood cultures and was classified as no focus or secondary to another focus if cultures from other body sites were either concomitantly negative or positive, respectively. Invasive infections were defined by the isolation of *Hafnia alvei *from a normally sterile body fluid or deep site.

### Statistical analysis

All analyses were performed using Stata version 9.0 (Stata Corp, College Station, TX). Differences in proportions among categorical data were assessed using Fisher's exact test. Medians with interquartile ranges (IQR) were used to describe non-normally distributed continuous variables. Incidence rates were calculated using regional demographic data as the denominator and compared using Poisson counts. Category specific risks were calculated and reported as risk ratios (RR) with exact 95% confidence intervals (CI) as previously described [9].

## Results

**Figure 1:**
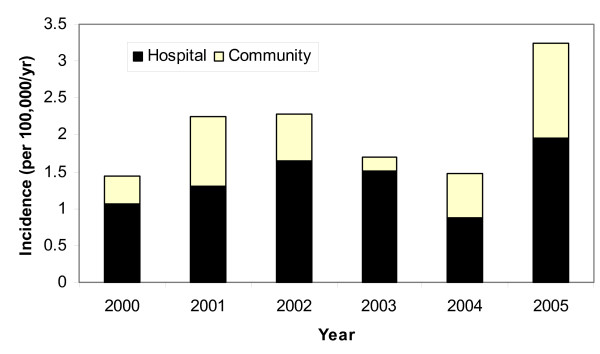
Annual incidence of hospital and community based isolation of *Hafnia alvei *in the Calgary Health Region, 2000–2005.

During the six years of surveillance, a total of 138 patients with a *Hafnia alvei *isolate were identified for an overall annual population incidence of 2.1 per 100,000 population. The annual incidence of isolation varied considerably during the years of the study as shown in Figure 1. There was no seasonal pattern of occurrence evident. The majority (93; 67%) were community isolates. Hospital isolates were first identified a median of 13 (IQR; 6–33) days after admission to hospital.

**Figure 2:**
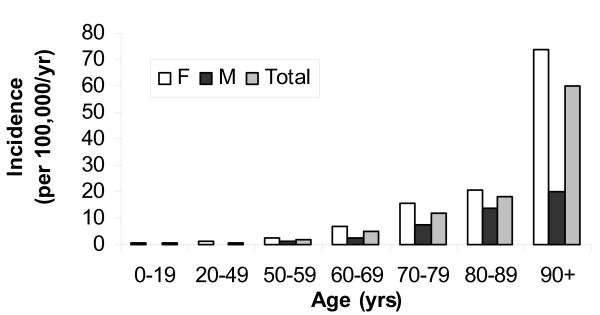
Age and gender specific incidence rates of *Hafnia alvei *isolation in the Calgary Health Region, 2000–2005 (F = Female; M = Male).

The median age of the patients was 69 (IQR; 49–79) years. There was a significant increase in the incidence associated with advancing age with the highest rate of 60 per 100,000 per year observed in those aged 90 years and older as shown in Figure 2. Overall 106 (77%) of the patients were female. The isolation rate was significantly higher among females as compared to males (3.2 vs. 1.0 per 100,000; RR 3.3; 95% CI, 2.20–5.07; p < 0.0001) overall, and this was most pronounced among those aged 90 years and older (Figure 2). As compared to females, males were much more likely to have hospital isolates (19/32; 59% vs. 26/106; 25%, RR 2.4; 95% CI 1.56–3.76; p < 0.001). Among the 19 hospital isolates in males, 8 were classified as urine, 7 lower respiratory, 2 soft tissue, and 1 each as intra-abdominal and bacteremia without focus. In contrast, in the 26 hospital isolates from women all but 3 were from urine; 2 were intra-abdominal and 1 was bacteremia without focus.

The most common focus of isolation was urine in 112 (81%), followed by lower respiratory tract in 10 (7%), and soft tissue in 5 (4%). The foci of isolation in association with other demographic and clinical variables are shown in Table 1. The annual incidence of non-urinary foci observed in females of 0.36 per 100,000 was comparable to the incidence in males of 0.42 per 100,000 (RR 0.85; 95% CI, 0.36–1.89; p = 0.7). Fourteen patients had invasive infections for an incidence of 0.21 per 100,000/year; nine of these were bacteremias (6 no focus and 3 urinary tract) and five were intra-peritoneal samples.

**Table 1:** Features of *Hafnia alvei *isolation according to focus.

**Source**	**Age (interquartile range)**	**Female**	**Hospital isolation**	**Polymicrobial etiology**
Urine (n = 112)	69 (46–80.5)	94 (84%)	31 (28%)	25 (22%)
Lower respiratory (n = 10)	64 (51–76)	2 (20%)	7 (70%)	7 (70%)
Soft tissue (n = 5)	61 (60–66)	3 (60%)	2 (40%)	4 (80%)
Bacteremia without focus (n = 6)	78.5 (74–84)	3 (50%)	2 (33%)	4 (67%)
Intra-abdominal (n = 5)	62 (47–73)	4 (80%)	3 (60%)	4 (80%)

In the majority of cases (94; 68%) *Hafnia alvei *was isolated from clinical samples as a mono-microbial etiology, and this was significantly associated with the focus of isolation (Table 1, p < 0.001). Non-urinary tract foci were more than three-times more likely to be poly-microbial as compared to the urinary tract isolates [19/26 (73%) vs. 25/112 (22%); RR 3.3; 95% CI, 2.16–4.97; p < 0.0001]. In the 44 patients who had polymicrobial cultures, the co-isolates included *Enterococcus *species in 17, *Escherichia coli *in 9, *Klebsiella *species in 6, *Staphylococcus aureus *in 4, *Pseudomonas aeruginosa *in 3, *Candida *species in 3, *Citrobacter *species in 3, group B streptococcus in 2, and 1 each of *Enterobacter cloacae*, *Haemophilus influenzae*, streptococcus milleri group, and *Bacteroides fragilis*.

The majority of *Hafnia alvei *isolates were resistant to ampicillin (111;80%), cephalothin (106; 77%), amoxicillin/clavulanate (98; 71%), and cefazolin (95; 69%). Lower rates of resistance were seen with cefpodoxime (56; 41%), gentamicin (12; 9%), cefuroxime (15; 11%), piperacillin/tazobactam (15; 11%), ceftazidime (9; 7%), ceftriaxone (8; 6%), nitrofurantoin (4; 3%), and trimethoprim-sulfamethoxazole (2; 1%). None of the isolates demonstrated resistance to either imipenem or ciprofloxacin. Overall 20 (14%) of isolates were susceptible to all the above anti-microbial agents tested. Female patients (19/106;18% vs. 1/32; 3% for males; p = 0.04), urinary tract focus (19/112; 17% vs. 1/26; 4%; p = 0.1), and community isolates (20/93; 22% vs. 0/42 for hospital onset, p < 0.001) were associated with culturing fully susceptible strains.

## Discussion

The findings of our study challenge many of the currently held beliefs surrounding the epidemiology of *Hafnia alvei *infections. The limited body of pre-existing literature is composed primarily of hospital-based case reports and series [2-6]. These in general report that *Hafnia alvei *most commonly infects men, that it typically causes disease in association with other organisms as a poly-microbial etiology, that it is a predominantly nosocomial pathogen, and that the respiratory and gastrointestinal tracts are the most common sites of isolation. In contrast, we demonstrated that in a non-selected population that these infections are typically mono-microbial in nature, are more common in females, and that the large majority are community-onset urinary tract infections. The dramatic difference seen in our study compared to others likely reflects at least in part the selection bias in those studies largely resulting from their restriction to patients hospitalized in university-based acute care centres. In comparison, we studied all *Hafnia alvei *isolated from patients in our region including from hospitals, physician's offices, clinics, nursing homes, and urgent care centres.

This study documents the rarity of infections due to *Hafnia alvei*, with an overall annual isolation rate of 2 per 100,000 population and an incidence of 0.18 per 100,000 for invasive disease. To put this in context, the rate of invasive *Hafnia alvei *disease is approximately 100-fold lower than that reported for invasive pneumococcal (20–22 per 100,000) and *Staphylococcus aureus *disease (28 per 100,000) [9-11], and 10-fold lower than invasive *Candida *species infections (2.9 per 100,000) [12]. In contrast, our rate of *Hafnia alvei *bacteremia is nearly twice that previously thought based on crude estimates from the United Kingdom [1]. The higher incidence of *Hafnia alvei *isolation observed among females as compared to males (Figure 2) was largely due the dramatically higher rate of urinary foci seen among females (Table 1). We did observe substantial variability in the yearly isolation of *Hafnia alvei *with a spike in 2005 (Figure 1). While some of this may reflect natural variability, the apparent increase in 2005 remains unexplained by our data. Further investigation using a molecular typing method such as pulsed field gel electrophoresis would be required to assess for the presence of a clonal outbreak in that year.

Although our study reports novel population incidence rates for *Hafnia alvei *isolation, there are a few methodological aspects of our incidence determinations that merit discussion. Despite the fact that our study was unlikely to miss any significant number of cases of culture positive disease occurring in the CHR because virtually all microbiology laboratory services were included in surveillance, we likely overestimated the occurrence of disease due to *Hafnia alvei *as a result of two potential reasons. First, because we did not have clinical data to confirm the clinical significance of cultures, we likely included a number of patients that were not truly infected but rather colonized [1]. However, this was not an issue as regards invasive disease as these are by definition rarely, if ever, contaminants. Second, we did not confirm residency status of cases in this study but rather assumed that patients who had Alberta Health numbers as outpatients or where admitted to hospital were residents. We have previously shown that inclusion of non-residents to be an important bias at least in population-based studies in critically ill patients where referral is common [13]. Based on our experience with a range of population-based studies in our region, we estimate that we may have included approximately 15–20% non-residents. It is important to note that each of the limitations noted in our study would have the effect of overestimation of the true incidence rate; in any case these data therefore demonstrate that *Hafnia alvei *infections are truly uncommon.

Prior case reports and series have noted that isolation of *Hafnia alvei *appears to occur frequently in association with co-morbid illnesses such as malignancies and chronic heart, liver, and lung disease [2,5]. Since the prevalence of co-morbid illnesses tends to increase with advancing age, our observations that the very old were at highest risk for isolating *Hafnia alvei *may provide indirect support for this. However, in order to define whether certain underlying conditions are actual risk factors for disease, knowledge of both the frequency of that factor among cases of disease in addition to the rate in a control population is required. No studies to date, including ours, have been adequately designed to establish risk factors for *Hafnia alvei *infection and further study is required.

Similar to the existing literature, we observed high rates of resistance to ampicillin, amoxicillin/clavulanate, cephalothin, and cefazolin, low rates of resistance to aminoglycosides, and second and third generation cephalosporins, and no isolates that demonstrated resistance to imipenem or ciprofloxacin [2,5,14]. *Hafnia alvei *is known to encode an AmpC-type β-lactamase that is distinct from many other Enterobacteriaceae that results in resistance to third generation cephalosporins [15,16]. Approximately one-quarter of our isolates were susceptible to ampicillin and cephalothin suggesting that these isolates do not produce an AmpC-type β-lactamase or do not express their enzymes in sufficient quantities to cause resistance.

In summary, we present the first population-based study investigating the epidemiology of *Hafnia alvei *infections and document that these infections are uncommon. We demonstrate that this organism is most commonly isolated from the urine of community-based patients and that females and the elderly are at highest risk. This study defines the distribution and determinants of *Hafnia alvei *isolation in a population at large and highlights the importance of population-based studies in accurately defining the epidemiology of an infectious disease.

## Abbreviations

Calgary Health Region (CHR)

Calgary Laboratory Services (CLS)

Confidence interval (CI)

Interquartile ranges (IQR)

Risk ratio (RR)

## Competing interests

The author(s) declare that they have no competing interests.

## Authors' contributions

KL conceived and designed the study, conducted the analyses, and drafted the manuscript. DC and TR were responsible for acquisition of data. JP contributed to study conception and design and interpretation of data. All authors contributed to and approved the final manuscript.
